# Level of Effort and Economic Dishonesty: Are Expectations Relevant?

**DOI:** 10.5964/ejop.10429

**Published:** 2023-11-30

**Authors:** Tomas Bonavia, Josué Brox-Ponce, María F. Rodrigo

**Affiliations:** 1 Faculty of Psychology, University of Valencia, Valencia, Spain; Università Cattolica del Sacro Cuore, Milan, Italy

**Keywords:** economic dishonesty, moral judgment, moral intuition, cheating behavior, expectations

## Abstract

Some research has shown that expectations modulate people’s economic dishonesty. These studies have allowed their participants to precisely establish the dishonest extra financial gain, without threatening their image of honesty. In this article, we show that in situations where our economic dishonesty is driven by hard-to-quantify motivators such as level of effort, it is difficult to change the categorization of (dishonest) judgments. Faced with this ambiguity, people make decisions guided by moral intuitions that are not conditioned by changing expectations. We carried out three studies (one single-group study and two experimental between-subjects studies) in which we tested whether the level of deception varies when manipulating expectations of transparency/privacy and dishonesty/honesty. Our results show that the levels of dishonesty remain low, regardless of the participants’ expectations. When our decisions are motivated by more ambiguous factors, in terms of being able to justify ourselves, our economic dishonesty becomes more rigidly directed toward the dictates of our moral intuitions.

Economic dishonesty has been found to have an extremely harmful global economic impact. The European Commission (2014) suggested that corruption costs EU countries 120 billion euros each year. This problem has not gone unnoticed by citizens around the world, given that, according to the Transparency International (2020) report, the perception of corruption remains high worldwide. In this context, understanding how economic dishonesty functions has become a particularly important topic in areas such as moral psychology, behavioral ethics, and economic psychology or behavioral economics. New contributions indicate that economic losses resulting from dishonesty are not just linked to a few bad apples, but rather they are the responsibility of a large number of people (Mazar & Ariely, 2015). In this direction, Ariely (2012) has argued that people tolerate cheating to the extent that they can obtain an economic benefit and retain a positive image of themselves. This approach highlights the predominance of our internal motivation system over external rewards (Mazar & Ariely, 2006).

This conception of economic dishonesty has been reintroduced in other studies that assess the impact of expectations on our decisions. Some research has proposed that the prevalence of breaking the rules can lead to high expectations of dishonesty (Mazar et al., 2008; Bicchieri, 2017), which can increase economic dishonesty (Lefebvre et al., 2015). In this regard, new strategies to combat economic dishonesty, such as transparency measures, are beginning to take root in today’s societies (Park & Blenkinsopp, 2011). However, experimental research that has addressed the impact of expectations has one common feature: it has been conducted under conditions where participants could obtain small extra economic benefits from being dishonest. For this reason, the aim of the present research is to examine the impact of expectations of honesty or dishonesty and privacy or transparency under conditions where people obtain the same economic benefit whether they cheat or not, and where the motivating factor for the deception is more ambiguous than obtaining an extra financial gain.

## Psychologically Limited Dishonesty

Throughout the years, great efforts have been made to propose behavioral models to explain dishonesty (see Jacobsen et al., 2018). One of the most promising models is the theory of self-concept maintenance (SCM) by Mazar et al. (2008). This model states that dishonesty is the result of the competition between two main motivations: the benefit we get from cheating and the need to maintain a positive self-concept in terms of honesty. Both factors reflect our tendency to harmonize our short-term self-interest with the desire to maintain a positive reputation in the long term (Gino, 2015). This balance results in acceptable levels of deception or in ethical dissonance (Barkan et al., 2015). People reduce ethical dissonance thanks to specific contextual factors, self-serving biases, and justifications that enable them to benefit from dishonesty without negatively updating their moral ledger (Ayal et al., 2021).

According to the SCM, the two basic processes underlying our dishonest decisions are categorization and attention to one’s internal standards of conduct (Mazar et al., 2008). Studies have shown that certain types of actions and magnitudes of deception are easier to categorize as dishonest. Mazar and Ariely (2006) believe that, in situations where the potential level of economic dishonesty is extremely low or extremely high, our decisions are governed by a cost-benefit analysis. However, as the potential economic dishonesty reaches a more moderate range, internal standards of behavior are activated. In addition, our tolerance level for cheating can be affected by various factors that Ariely (2012) groups into two classes. Some of the factors that increase tolerance to cheating are the ability to rationalize or engage in self-deception, the exposure to conflicts of interest, creativity, past immoral actions, feeling depleted, a contagion effect from watching others behave dishonestly, and living in a dishonest culture. Some of the factors that reduce tolerance to deception would be moral reminders, pledges, and supervision. The method used to show how these factors and magnitudes affect people’s economic dishonesty has consisted of finding out how much extra money they are willing to obtain by cheating (see Ariely, 2012). However, this methodology has neglected situations where economic dishonesty may be mainly motivated by factors other than obtaining extra financial gain. Thus, these types of observations involving economic gain should be complemented by others where factors other than economic profit can be the main reason for tolerance to deception. This approach would allow us to understand that the expected benefit mentioned in the SCM does not have to be exclusively economic. Instead, it can be linked to the internal preferences of the decision makers based on other variables such as: the level of effort, the intrinsic attraction of the activity, luck, its difficulty, or familiarity with it.

In addition, in the case of attention to internal standards of behavior, the SCM has not sufficiently addressed the role of emotions in this process. This question has been widely examined by authors who study the role of moral intuitions in judgment and decision making (Sinnott-Armstrong et al., 2010), such as Jonathan Haidt (2001) and his social intuitionist model (SIM). The central thesis of the SIM is that moral judgments are the product of intuitions, followed by a posteriori reasoning. The latter are understood as justifications or rationalizations for the previous unconscious moral assessment (Haidt, 2013). As the main source of our moral intuitions, Haidt (2003) points to moral emotions such as guilt, shame, disgust, anger, contempt, compassion, gratitude, and elevation. The way these moral emotions allow a variety of moral views to exist throughout the world is explained by the impact of cultural learning and socialization on an evolutionary moral structure common to our species (Graham et al., 2013). For this reason, our dishonest judgments are strongly conditioned by affective elements that escape our conscious level. A similar approach is followed by Nichols (2004), who understands that judging the correctness of an action, understood as a transgression of a sentimental rule, involves knowing a normative theory about the action and an affective mechanism that highlights its prohibitive nature. Thus, when we lie, for example, we are aware of our lack of ethics because we have the norm that prohibits it and the emotional activation that makes this transgression important.

Emphasizing the role of intuitions in our moral judgments does not rule out the existence of all forms of deliberation. Greene's (2013) studies show that our moral judgements are the product of two competing and interacting psychological systems. One is based on automatic emotional responses, and the other is based on conscious deliberation (Bereby-Meyer et al., 2020). The activity of these two systems takes place in different neural areas (Greene et al., 2004): the former in the amygdala, anterior cingulate cortex, ventromedial prefrontal cortex, and posterior superior temporal sulcus; and the latter in the dorsolateral prefrontal cortex and bilateral inferior parietal lobe. Consequently, the two systems involve different emotional processes (Cushman et al., 2010). The system of emotional automatic settings uses emotional alarms that provide definitive demands and restrictions, whereas the controlled cognition system offers negotiable motivations that are inserted into our practical reasoning (Hatta et al., 2022). This proposal makes it possible to understand why some moral judgments show a certain degree of unconditionality to social factors, given that some behaviors activate the alarm system and are strongly rejected by our automatic emotional responses (moral intuitions). Hence, moral emotions can function as (dis)honesty markers that direct our decisions to morally acceptable positions. This mechanism can explain that we have a window of tolerance for deception where we are relatively insensitive to external rewards (Mazar & Ariely, 2006). Furthermore, it also explains that certain (dis)honest behaviors are unaffected by the influence of social factors, due to the activation of moral intuitions that do not alter the immoral consideration of an action. Of course, this does not mean that certain social and situational elements do not condition our level of deception. It simply introduces the need to explore in what situations they do or do not facilitate tolerance to deception. Therefore, in this paper, we will investigate how certain types of expectations influence the level of economic (dis)honesty.

## Expectations of (Dis)Honesty

Bicchieri (2006, 2017) identifies two key concepts in discriminating between group behavioral patterns: preferences and expectations. She defines preferences as individual or social dispositions to act in a certain way in a specific situation. Among the social dispositions, she differentiates between preferences conditioned by the expectations of others and those based on unconditioned social comparisons. In sum, she states that conditioned preferences are determined by empirical expectations (what we expect others to do) and normative expectations (what we expect others to think about what we should do). According to Bicchieri and Xiao (2009), both types of expectations affect our choices, but empirical expectations have the most influence. This conclusion is quite interesting if we transfer it to the field of economic dishonesty because, according to Bicchieri (2017, p. 86):

The updated empirical expectations easily bleed into the normative realm. Disclosing information about how common some “bad” behaviors are is counterproductive. Think of illegally downloading music or television shows. It is rampant, and people vaguely have the feeling that it is wrong, but at the same time, everyone does it. The same goes for bribing or violent behaviors when they are pervasive. We know these behaviors are condemned, but when empirical and normative expectations diverge, people are tempted to follow the crowd, because the normative message loses power.

Through this idea, C. Bicchieri suggests that empirical expectations can condition dishonest people. This issue has not gone unnoticed, as some research shows. Dong et al. (2012) propose the concept of “conditional corruption” to refer to situations in which people see their corrupt behavior conditioned by the behavior of others. These authors offer a large amount of data that indicate that people's propensity to display corrupt behavior depends on the quality of the institutions and the level of corruption they perceive in their social environment. They view corruption as a cooperative behavior that arises from the reduction in the moral and legal costs stemming from certain transgressions. Along these lines, Gächter and Schulz (2016) showed that societies that are legislatively weak and culturally permissive about breaking the rules condition the intrinsic honesty of their citizens. Similarly, Falk and Fischbacher (2002) found that people's theft behavior is affected by the level of theft inherent in their environment. This finding led them to use the term “conditional norm violators” to describe people who stole more due to the high levels of theft around them. In addition, Lefebvre et al. (2015) found that people made the decision to evade paying taxes more when they were informed that there had been a generalized low tax compliance (this effect was not replicated when the expectation conveyed a generalized compliance).

Together, these approaches reveal that empirical expectations of economic dishonesty affect our decisions in particular circumstances, that is, when the main motivating factor is extra economic benefit and, by extension, when we can precisely determine our level of dishonesty. Most of the experiments carried out on expectations and dishonesty have consisted of allowing the participants to obtain small extra economic benefits from being dishonest (Bicchieri 2006, 2017; Mazar & Ariely, 2006, 2015). We believe that factors other than economic benefit create more ambiguity for the decision maker about the acceptable level of economic dishonesty. In addition, given that we are often forced to make an exclusive choice between being dishonest or not, (dis)honest decisions can acquire a certain degree of unconditionality with regard to our expectations. It is clear that expectations have been shown to be a key factor in modulating cooperation and self-interest in our economic decisions (see Bicchieri & Chavez, 2010), but it is important to remember that dishonesty involves cheating, deception, and other types of unethical behavior that challenge the general interest of society (Gino, 2015). For this reason, this type of behavior involves infringing norms that produce strong ethical dissonance, which crystallize into a decision that cannot easily be modified by social factors (Barkan et al., 2015; Bicchieri, 2017; Turiel 1983). Thus, although expectations have an impact on economic dishonesty in certain conditions, it is difficult to imagine that this effect can be extended to all kinds of situations. Therefore, it is important to clarify when decisions driven by internal standards of behavior are robust enough to be unconditioned by our expectations.

## Transparency as Expectation

Transparency measures have also been suggested to influence dishonesty levels. In general, we can define transparency as open flow and public access to information (Kolstad & Wiig, 2009). In the context of decision making, a transparent decision can be understood as a decision that is made public to others. The possibility of unethical decisions being made public has been found to produce anticipated feelings of shame in the decision maker, leading him/her to make ethical decisions (Bonavia & Brox-Ponce, 2018). This effect occurs because people try to avoid having their social image affected by behaviors that are rejected by others (Leary, 2005). Thus, people avoid making decisions that may be disapproved of by others, in an attempt to offer a positive image of themselves.

The transparency of economic decisions has been studied from microeconomic and macroeconomic perspectives. On the one hand, most macroeconomic research reveals a negative correlation between levels of transparency and levels of corruption. Specifically, transparency has been found to reduce corruption by helping to maintain integrity and trust in people (Park & Blenkinsopp, 2011). However, it has been noted that transparency requires individuals to have the necessary resources and skills to process and access this information (Lindstedt & Naurin, 2010). On the other hand, in the microeconomic field, some studies have explored the isolated impact of transparency on certain economic decisions (see Goulart et al., 2015). In these cases, the effect of transparency has been implemented by warning that the decisions would be made public to others, as in the case of an expectation. Based on this approach, Bonavia and Brox-Ponce (2018) showed that transparency has a moderate impact on reducing economic dishonesty in a situation where the options have unequal economic benefits. Participants in their experiment could choose between honestly or dishonestly obtaining a sure or likely economic gain of a given amount. In this way, participants could assess whether the economic benefit they obtained from being dishonest justified their deceitful behavior. Thus, the categorization of decisions as (dis)honest was simpler, and, therefore, transparency had a slight impact on the decision-making process.

Beyond the studies mentioned above, little is known about how the expectation of transparency can affect our level of economic dishonesty when it is motivated by factors that are quantitatively more ambiguous than the pursuit of economic gain. In this regard, analogous to the case of expectations of economic (dis)honesty, we might suspect that the deterrent effect of transparency is associated with experiments in which people can determine precisely how much money they can steal.

## Objective and Hypotheses

According to the SCM, the core of economic dishonesty consists of the tension between the motivation for economic gain and the desire to maintain a positive self-concept in terms of honesty. The possibility of establishing extra financial benefits facilitates the process of categorizing a decision as (dis)honest. In turn, it allows expectations about others’ behavior to influence our level of tolerance for deception, given that small leaps in dishonesty are morally acceptable to the decision maker. However, more ambiguous motivators can impede the categorization process and its moral justification. Thus, people would be guided more by intuition or internal standards of behavior than by expectations about others’ behavior. Given the aforementioned arguments, the main objective of this paper is to test the effect of expectations of honesty or dishonesty, and transparency or privacy on people's (dis)honest behavior when the same economic benefit can be obtained. We aim to clarify the impact of these expectations on our decisions when they are based on difficult-to-quantify motivators such as the level of effort. We believe that under certain conditions, (dis)honest decisions reach a higher degree of unconditionality regarding the behavior we expect from other people.

In the following three studies, the dependent variable is the decision (dishonest or honest) made by the participants when confronted with a dilemma. This is a dichotomous variable, so that the participants must choose between two options: a) the dishonest option (to gain 300 euros from a grant that does not correspond to him/her), and b) the honest option (to gain 300 euros from performing a job). The first option represents gain without effort, and the second represents gain with effort. Study 1 consists of a single group design. Studies 2 and 3 are one-way between-subjects designs. The independent manipulated variables are expectations of transparency (public vs. private decision) in Study 2 and expectations of honesty or dishonesty in Study 3.

The proposed hypotheses for the three studies are:

**H1**: People prefer to be honest if they can choose between options with the same economic value, even if it requires more effort (Study 1).
**H2**: People prefer to be honest if they can choose between options with the same economic value, even if it requires more effort and their decision is private to others (Study 2. This hypothesis is a more specific version of H1, where participants are informed that their decision would not be known to anyone.
**H3**: Public and private expectations will have an effect on the decision, so that the public expectation will lead to a greater number of honest decisions (Study 2).
**H4**: People prefer to be honest if they can choose between options with the same economic value, even if it requires more effort and there are empirical expectations of dishonesty (Study 3). This hypothesis is a more specific version of H1, where the participants are informed that most people had chosen the dishonest option in a previous experiment.
**H5**: The empirical expectations of honesty and dishonesty will have an effect on the decision, so that the empirical expectation of honesty will lead to a greater number of honest decisions (Study 3).

Hypotheses 1, 2, and 4 try to offer support for the same basic idea: motivators that are more ambiguous than extra economic benefit make it difficult to categorize a decision as dishonest and, as a result, block the effect of expectations. To test this, the decision-making problems offer the possibility of obtaining the same economic gain, either through a dishonest decision that does not require effort or through an honest one that does. Therefore, our choice will depend on our preference to maintain a positive image of ourselves or, on the contrary, to obtain the same reward for less effort. This gives the dishonest choice a certain attraction, and some level of dishonesty is reached, which makes it possible to compare groups of participants. Obviously, the level of effort is not as easily quantifiable as the extra economic benefit commonly used in experiments (Ariely, 2012). Thus, given that effort is a more ambiguous motivator, we expect the decision process to be determined by moral intuitions that function as moral markers of dishonesty because the decision maker will not be able to establish a specific level of deception that is morally acceptable to him/her. Hypotheses 3 and 5 are derived from the literature review and focus on comparing the different expectations, so that both the expectations of transparency and the expectations of honesty will have the effect of increasing the choice of the honest option.

## The Use of Hypothetical Scenarios in Research on Dishonesty

Hypothetical scenarios are widely used in social science research and, more specifically, in the study of dishonesty. The hypothetical scenario method is commonly used to study offender decision-making, and it is fundamental in a wide range of criminology and criminal justice research (Exum & Layana, 2018). Much of the research on morality and ethical judgments has used the scenario-based approach (McMahon & Harvey, 2007). These scenarios have also been used to study organizational factors associated with dishonest employee behavior in the retail sector (Jaakson et al., 2017), as well as in other research, for example, to find out what motivates consumers to lie (Argo et al., 2006), a person’s reasons for deception (Cassidy et al., 2019), or why students plagiarize or engage in other forms of cheating (Waltzer & Dahl, 2021). Thus, to measure dishonest behavior, researchers often use scenarios because they can accurately reflect individuals’ emotions, intentions, and behaviors in different situations (Wenzel & Reinhard, 2020).

The main limitation of using scenarios is that we cannot be sure that participants are sincere about their true inclination to act honestly or dishonestly. People who say they would behave unethically in a hypothetical situation might change their minds if they were in the real situation. Likewise, when faced with situations that pose a moral dilemma, we might imagine that it is easier for a person to indicate that s/he would behave honestly because, in reality, s/he has nothing to gain or lose. However, according to Argo et al. (2006, p. 107), “although scenario-based methodology has limitations, this approach may have provided a more stringent assessment of lying intentions because real situations are more involving, and the propensity to lie in such situations is likely to be higher than in response to scenarios”.

Currently, some studies successfully combine both procedures, hypothetical scenarios and real situations, to research dishonesty (see Shu et al., 2011). Simulated situations (such as a mock job interview), which would be halfway between a hypothetical scenario and a real situation, have also been used to determine when a person will lie or tell the truth (Walczyk et al., 2016). These investigations, as in all the studies cited above, except one (Exum & Layana, 2018), confirm that the results found in hypothetical situations can be used as proxies for real life.

Finally, one of the advantages associated with the use of hypothetical scenarios is that they can be adapted to the type of participants who have to respond. In our case, because the sample consisted of undergraduate students, the scenarios were designed within an academic context to promote a higher level of engagement with the proposed situations (Masip et al., 2016).

## Studies 1 and 2: Effect of Expectations of Transparency and Privacy

Study 1 tests whether people prefer to be honest if they can choose between options with the same economic value, even if it requires more effort for them. In Study 2, we experimentally assess whether the introduction of privacy and transparency expectations affect the dishonesty levels of the participants (H2 and H3). To test this, as in the investigation by Bonavia and Brox-Ponce (2018), some participants were informed that their decision would be made public to others (expectations of transparency), and the rest were told that their decision would not be known to anyone (expectations of privacy). The basic proposal is that these expectations do not significantly influence the participants’ levels of economic dishonesty under these conditions. Given that both options involve the same economic gain, our decisions have to be conditioned by factors that offer greater ambiguity in justifying our deception (because we cannot precisely quantify a preference based on the level of effort). This situation, along with the fact that the decisions are mutually exclusive, introduces the possibility that it is more difficult to justify our image of honesty if we choose the dishonest option.

### Method

The procedure followed was exactly the same in the three studies described below. University students (all over 18 years old) were asked to participate voluntarily in these experiments. Before entering the room where the experimental procedure was carried out, all the students were informed of the conditions for participating in each experiment, and they were told that they could stop filling out the forms at any time. Right before entering the room, their verbal consent was obtained to continue with the experiment. The privacy and anonymity of their answers were always guaranteed.

At the beginning of each experiment, they received a sheet of paper with a decision problem. Participants were told that, after reading the statement carefully, they should choose one of the options (or not answer, leaving the form blank if they did not want to participate in the study), and they were informed that there were no right or wrong answers.

In addition, approval was received from the Ethics Commission on Experimental Research at the University where the study took place. This commission is governed by the guidelines stipulated in the Helsinki Declaration.

Study 1 consists of a single group design. The experiment in Study 2 has a one-way between-subjects design with two conditions (privacy and transparency expectations). Specifically, 39 students from a Spanish university participated in Study 1, and a total of 79 in Study 2. In both studies, the majority of the participants were 24 years old or younger (85%); the mean age was 22.92 (*SD* = 6.21, range = 35) in Study 1 and 23.45 (*SD* = 6.67, range = 40) in Study 2. There was only one group in Study 1, consisting of 6 men (15.4%), and 33 women (84.6%). Two independent groups of participants were randomly assigned to the two experimental conditions in Study 2; the privacy expectation condition had 40 participants (8 men and 32 women, 20% and 80% respectively), and the transparency expectation condition had 39 participants (7 men and 32 women, 17.9% and 82.1% respectively). A chi-square test was performed to determine whether the proportion of women was equal in the two experimental conditions in Study 2. The proportion of women did not differ by condition, χ^2^ (1, *N* = 79) = 0.05, *p* = .82.

In Study 1, the following introductory statement was presented: “Please imagine that you have to choose between the two options presented below”. For each experimental condition in Study 2, the following introductory statement was presented:

- Expectation of privacy group: “Please imagine that you have to choose between the two options presented below. Taking into account that your decision will never be known by anyone, please mark which one you would choose”.
- Expectation of transparency group: “Please imagine that you have to choose between the two options below. Taking into account that your decision will be made public and known by the whole world, please mark which one you would choose”.

Similar quantities and response options to those of Bonavia and Brox-Ponce (2018) were used in both studies. Thus, choosing option (a) involved receiving a dishonest financial gain (receiving a scholarship that does not correspond to you), and option (b) involved receiving an honest economic benefit (performing a routine job organizing documents in the faculty administration). Both options had the same financial gain: 300 Euros; but they involved a very different level of effort: the first option represents gain without effort, and the second represents gain with effort. Literally, the participants chose one of the two response options:

- A 300 € gain due to receiving a grant that does not correspond to you.
- A 300 € gain from performing a routine job organizing documents in an administration office of the faculty.

To test H1 (Study 1) and H2 (Study 2) a binomial test was performed to evaluate whether the proportion of honest decisions was equal to 0.5. To test H2, only the data under the privacy condition, in Study 2, were considered. To test H3 (Study 2), we performed a chi-square test (χ^2^) to test the effect of public and private expectations on the number of honest and dishonest decisions. In addition, the effect size was evaluated through the Cramer V test. All the statistical analyses, both for these studies and the following one, were performed with the SPSS 22 statistical analysis program.

A priori sample size analysis was performed using G*Power (Version 3.1.9.7; Faul et al., 2007) to determine the sample sizes in Studies 1 and 2 to ensure a power of 0.80. In Study 1, the a priori power analysis showed that a sample of *N* = 30 would be enough to ensure a power of 0.80, with an α = .05, to detect an effect size of *g* = 0.25, in a binomial test. In Study 2, a sample of *N* = 50 would be sufficient to detect a medium to large effect size (w = 0.40) in a chi-square test. To avoid possible incomplete data or other problems (non-attending participants, etc.), we decided to oversample.

### Results

In Study 1, 30 participants (77%) chose the honest option, and 9 participants (23%) chose the dishonest option. The binomial test compared the proportion of honest decisions with *p* = .50 and the result was statistically significant (*p* = .001).

In Study 2, 67.5% vs. 32.5% of the participants in the private condition chose the honest option (see Table 1). The binomial test showed, as stated in H2, that the proportion of honest decisions was statistically higher than 0.50 (*p* = .038). As for H3, the effect of privacy and transparency expectations on the number of honest and dishonest decisions was statistically significant ($\chi^{2}=5.785,\;$*p* = .016) with a medium effect size (Cramer’s V = 0.27), so that the percentage of honest decisions increased from 67.5% to 89.7% in the condition of public or transparency expectations.

**Table 1:** Frequency Distribution of Answers for Both Privacy and Transparency Groups (Study 2)

	Options		
Group	(a) dishonest	(b) honest	Total *n*
Privacy group	13 (32.5%)	27 (67.5%)	40 (100%)
Transparency group	4 (10.3%)	35 (89.7%)	39 (100%)
Total	17 (21.5%)	62 (78.5%)	79 (100%)

The results of Study 1 and Study 2 confirm H1, H2, and H3. The participants preferred to choose the honest alternative, even though the dishonest alternative allowed them to obtain the same economic benefit without any effort. This result was replicated (in Study 2), even when their decision was not known to others. Moreover, expectations of transparency or privacy had a significant effect on the decisions, so that the number of dishonest decisions decreased in the condition of public or transparency expectations.

## Study 3: Effect of Expectations of Honesty and Dishonesty

In Experiment 3, we tried to discover how expectations of (dis)honesty affect the participants’ decisions (H4 and H5). Contrary to the results of Lefebvre et al. (2015), we expected that participants would feel compelled to choose the honest option, even if there are empirical expectations of dishonesty. As in Experiment 2, this unconditionality is expected because there is no possibility of specifying an extra economic benefit. In these conditions, the participant had to choose between being completely honest or dishonest while assessing the level of effort required to obtain the money. For this reason, the unconditionality that characterizes certain moral judgments (Bicchieri, 2017) can operate as a mechanism to contain the effect of expectations of (dis)honesty.

### Method

This experiment has a one-way between-subjects design with two conditions (dishonesty and honesty expectations). Two independent groups of participants were randomly assigned to the two experimental conditions (*N* = 83). The mean age was 22.67 (*SD* = 3.61, range = 19). The expectations of dishonesty condition contained 42 participants (15 men and 27 women, 35.7% and 64.3%, respectively), and the expectations of honesty condition had 41 participants (13 men and 28 women, 31.7% and 68.3%, respectively). A chi-square test was performed to determine whether the proportion of women was equal in the two experimental conditions. The proportion of women did not differ by condition, χ^2^ (1, *N* = 83) = 0.15, *p* = .70. A priori power analysis using G*Power (Version 3.1.9.7; Faul et al., 2007) showed that a sample of *N* = 50 would be enough to ensure a statistical power of 0.80, with an α = .05, to detect a medium to large effect size (w = 0.40) in a chi-square test. To avoid possible incomplete data or other problems (non-attending participants, etc.), we decided to oversample.

To test Hypotheses 4 and 5, one group was informed that most people had chosen the dishonest option in a previous study (expectations of dishonesty), and the other group was told that there had been a prevalence of honest decisions (expectations of honesty). The introductory statements to the expectations were selected followed the guidelines established by C. Bicchieri, based on introductory questions used in other experiments that sought to assess the impact of empirical expectations on decision making (see Bicchieri & Chavez, 2010; Bicchieri & Xiao, 2009). The following are the introductory statements for each experimental condition:

- Expectation of dishonesty group: “Please, imagine that you have to choose between the two options presented below. Taking into account that 60% of the people who participated in this experiment last year chose option (a) instead of (b), mark the one you would choose”.
- Expectation of honesty group: “Please, imagine that you have to choose between the two options presented below. Taking into account that 60% of the people who participated in this experiment last year chose option (b) instead of (a), mark the one you would choose”.

The same response options were used as in Studies 1 and 2. Analogous to Study 2, H4 and H5 were tested by binomial and chi-square tests (${\text{χ}}^{2}$), respectively. To test H4, only the data for the dishonesty expectations condition were considered.

### Results

Regarding H4, in the condition of dishonesty expectation, 73.8% of the participants chose the honest decision, and 26.2% chose the dishonest decision (see Table 2). The binomial test compared the proportion of honest decisions with *p* = .50 and the result was statistically significant (*p* = .003). Regarding H5, the effect of empirical expectations of honesty or dishonesty was not statistically significant ($\chi^{2}=0.307,\;$*p* = .579).

**Table 2:** Frequency Distribution of Answers for Both Dishonesty and Honesty Groups (Study 3)

	Options		
Group	(a) dishonest	(b) honest	Total *n*
Dishonesty group	11 (26.2%)	31 (73.8%)	42 (100%)
Honesty group	13 (31.7%)	28 (68.3%)	41 (100%)
Total	24 (28.9%)	59 (71.1%)	83 (100%)

In summary, these results support H4, so that the number of honest decisions is superior to dishonest decisions, even under empirical expectations of dishonesty. In contrast, H5 is not supported, so that empirical expectations of honesty/dishonesty do not have an effect on the number of honest and dishonest decisions.

## General Discussion

Although the research on economic dishonesty has advanced in the past decade, a coherent theoretical framework that accounts for the social components of deception has not yet been achieved (Jacobsen et al., 2018). The SCM (Mazar et al., 2008) is a promising model, as shown in several experiments (see Gino et al., 2009). It mainly places the origin of dishonesty in the tension between obtaining an economic benefit and maintaining a positive self-concept in terms of honesty. The resulting window of tolerance for deceit can be affected by various factors (Ariely, 2012). One of the factors studied the most is the contagion effect of expectations of dishonesty, which is closely related to approaches such as conditional corruption (Dong et al., 2012), conditional norm violators (Falk & Fischbacher, 2002), or culturally permissive societies with regard to norm violators (Gächter & Schulz, 2016). These theories share a common principle: people's economic (dis)honesty can be affected by their expectations about others’ behavior (Bicchieri, 2006, 2017). However, this principle has been tested under experimental conditions that favor the effects of expectations. Specifically, people could establish the exact economic amount for which they would be dishonest (see Ariely, 2012; Lefebvre et al., 2015).

For this reason, we conducted three studies that offered the possibility of obtaining the same economic gain, either through a dishonest decision that does not require effort or through an honest one that does. Our results show that most people take honest decisions if they can choose between options with the same economic value, even if it requires more effort (H1, Study 1). This result was replicated under unfavorable conditions: private decision (H2, Study 2) and empirical expectations of dishonesty (H4, Study 3). Moreover, the manipulation of private/public decisions had an effect on the number of honest decisions (H3, Study 2), but the effect of the expectations of dishonesty/honesty was not statistically significant (H5, Study 3). Future work could consider the effect of both variables (expectations of transparency and expectations of dishonesty/honesty) on the decision simultaneously through a two-factor experimental design.

In summary, the participants’ levels of economic dishonesty remained low when different types of expectations were introduced. The experimental conditions involved having to exclusively choose between dishonest or honest behavior when there was a fixed economic amount. The main motive for economic dishonesty in these studies was to obtain easy money without effort (through a grant that did not correspond to the person who answered). We interpret this to mean that, when people are unable to establish a specific level of dishonesty, they tend to make decisions guided by pre-determined intuitions about what is right. As the SIM proposes (Haidt, 2013), a morally reprehensible event triggers an automatic intuitive reaction in people that guides the decision-making process. These reactions can be mitigated when we can determine a level of deception that is compatible with maintaining a positive self-concept; in other words, the benefit obtained is sufficient reward to justify a transgression that we consider minor (Barkan et al., 2015; Mazar et al., 2008). However, these small transgressions are possible in situations where we may need a specific economic benefit. Our research shows that in cases where the benefit is related to rewards that are difficult to quantify (level of effort), people’s judgments are anchored in pre-determined moral intuitions. As a result, the process of categorizing an action as dishonest is less susceptible to fluctuations in our expectations, which indicates that our moral judgments sometimes acquire a certain degree of unconditionality (Bicchieri, 2017; Turiel, 1983).

Our results point in one direction: when our decisions are influenced by more ambiguous factors in terms of justifying our decisions, our economic dishonesty is more rigidly directed towards the dictates of our internal standards for behavior (and expectations, ours and those of others, have much less influence). This conclusion highlights the need to identify which specific circumstances and behaviors block the impact of our expectations on our levels of economic dishonesty. This task is quite difficult because dishonesty as a moral behavior depends on cultural socialization processes (Haidt, 2013). However, to mitigate deceit effectively, it is essential to have a rational construction of what we understand as dishonesty (Bicchieri, 2006) and obtain experimental information about culturally pre-determined contextual circumstances and behaviors.

In fact, a limitation of our studies is that dishonest behavior is not free of cultural biases. In order to extrapolate these results to other groups of people and dishonest behaviors, it is necessary to use decision problems that are not affected by the context, such as the matrices employed by Ariely (2012). Of course, the two approaches are complementary because we need objective decision-making problems, but also problems that are specific to people's natural environment. Future studies will have to more deeply examine how to work with expectations for higher or lower economic rewards. The function that relates the activation of our internal motivational system to the variation in the extra financial reward was proposed by Mazar and Ariely (2006), but not in cases where financial gain is not the driving force behind economic dishonesty. This could be one of the next steps in advancing the understanding of the social components of deception.

Another consideration is that, in the three studies in this work, the proportion of women was higher than the proportion of men. Sex was not a confounding variable in Study 2 and Study 3 because the proportion of women and men was equal in the two experimental conditions in each study. The role of sex as a predictor variable of honest or dishonest decisions was not the focus of this work, but it should be addressed in future research.

From a practical point of view, efforts to make people's economic dishonesty transparent and expose their levels of corruption are not the best strategies for the type of behavior analyzed in these studies (although Hypothesis 3 has been confirmed). Extra economic benefit is not always the driving force behind our economic dishonesty, and so we also need to understand behaviors that lead to savings at the level of effort. Many people do not work because they are able to obtain enough money through dishonest effort-saving behaviors. Some examples are the illegal sale of all kinds of products (music, drugs, counterfeiting...), undeclared work, or professional intrusion. These people choose to deceive, even though the economic gain obtained may not be greater than what they would achieve by making a morally correct effort. Moreover, they are aware of their decision. According to our results, this type of economic dishonesty requires different measures, probably at the level of previously consolidated moral intuitions. These intuitions are shaped throughout the life cycle in all kinds of contexts, informing us about what is right, but sometimes they are ignored in favor of the benefit we hope to obtain. We think it is necessary to act in two directions: invest in ethical-civic education that makes it possible to reinforce the power of intuitions and moral reasonings that avoid economic dishonesty; and create complementary intuitions that highlight the economic value of honesty in the long term. These latter intuitions can dissuade people from the short-term profit that greatly harms today’s societies. In short, in order to choose intervention strategies that effectively reduce the level of deceit, it is necessary to first know the nature of the main motivator for each specific economic behavior. Not all dishonest behaviors are equal.
